# Transcript and blood-microbiome analysis towards a blood diagnostic tool for goats affected by *Haemonchus contortus*

**DOI:** 10.1038/s41598-022-08939-x

**Published:** 2022-03-30

**Authors:** Yonathan Tilahun, Jessica Quijada Pinango, Felicia Johnson, Charles Lett, Kayla Smith, Terry Gipson, Malcolm McCallum, Peter Hoyt, Andrew Tritt, Archana Yadav, Mostafa Elshahed, Zaisen Wang

**Affiliations:** 1 School of Agriculture and Applied Sciences, Langston University, Langston, OK 73050 USA; 2 Institute for Biosecurity and Microbial Forensics, Oklahoma State University, Stillwater, OK 74078 USA; 3 Computational Research Division, Lawrence Berkeley National Laboratory, Berkeley, CA 94720 USA; 4 Department of Microbiology and Molecular Genetics, Oklahoma State University, Stillwater, OK 74078 USA

**Keywords:** Biological techniques, Biotechnology, Cell biology, Computational biology and bioinformatics, Developmental biology, Drug discovery, Genetics, Microbiology, Molecular biology, Systems biology, Environmental sciences, Biomarkers, Diseases, Health care, Health occupations, Medical research, Molecular medicine, Pathogenesis, Signs and symptoms

## Abstract

The Alpine goat (*Capra aegagrus hircus*) is parasitized by the barber pole worm (*Haemonchus contortus*). Hematological parameters from transcript and metagenome analysis in the host are reflective of infestation. We explored comparisons between blood samples of control, infected, infected zoledronic acid-treated, and infected antibody (anti-γδ T cells) treated wethers under controlled conditions. Seven days post-inoculation (dpi), we identified 7,627 transcripts associated with the different treatment types. Microbiome measurements at 7 dpi revealed fewer raw read counts across all treatments and a less diverse microbial flora than at 21 dpi. This study identifies treatment specific transcripts and an increase in microflora abundance and diversity as wethers age. Further, *F*/*B* ratio reflect health, based on depression or elevation above thresholds defined by the baseline of non-infected controls. Forty Alpine wethers were studied where blood samples were collected from five goats in four treatment groups on 7 dpi and 21 dpi. Transcript and microbiome profiles were obtained using the Partek Flow (St. Louis, Missouri, USA) software suites pipelines. Inflammation comparisons were based on the *Firmicutes*/*Bacteriodetes* ratios that are calculated as well as the reduction of microbial diversity.

## Introduction

*Haemonchus contortus* can prove fatal if untreated in cattle, sheep, and goats^1^. Circumstances increase susceptibility in goats up to eight weeks after parturition^2^. Even moderate infection by these nematodes can reduce milk production and lead to stunted growth in goats. Nematode egg counts are the primary method to diagnose infection before localized production losses^3^. Significant blood loss leads to visual signs of infestation that may be confused with or due to a combined effect of other types of parasitic diseases.

*Haemonchus contortus* produce excretory and secretory effects that reduce the host immune response^4,6^. Among the suite of host immune system responses, two very important tools are the host genome and microbiome components^5^. The specific methods that are used to avoid host surveillance are still under study, but one theory suggests helminth inflammation inhibition is completed by modulating butyrate biosynthesis^7,9^. Natural microbiotas provide resources for innate immunity^6,8^. This typically happens via altered diversity of microbiota when compared to un-infected animals^9^.

Transcripts often indicate how and when response to a stimulus or stimuli occur. The microbiome modulates responses via transcripts activated by internal and external environmental cues^10^. Therefore, an infestation of a host by a parasite that interacts with surrounding environmental factors causes the formation of an intricate web of stimuli and responses^11^. A tri-directional interaction is predicted^12^, whereby the host depends on its own genome and its vast microbiome content to defend itself against an external stressor such as parasite intrusion.

It is postulated that differences exist in the microbiome composition displayed among uninfected (controls), infected (treated), infected subsequently treated with zoledronic acid (ZA), and infected subsequently treated with anti-γδ T cell antibodies (AB). The control group and different treatments will be used to construct an observable quantitative range for “health” in wethers. The results of this study will lead towards the provision of a quantitative, non-invasive method of measurement using blood^1,11,14–20,30^.

As previously mentioned, *Haemonchus contortus* produce excretory and secretory effects that reduce the host immune response^4^. This is often the case, where a large array of exudates from intestinal helminths modulates microbial communities^6,8^. In addition, *H. contortus* competes with naturally occurring flora of the host for energy-rich nutrients or essential minerals^13^. Infection by *H. contortus* is known to impact intestinal physiology by increasing fluid secretions that alter the habitat of healthy bacterial communities^14^. In this study, we examine if infection by *H. contortus* impacts blood-based transcripts and/or diversity/richness of bacterial flora in wethers under different treatment conditions. Thereby indicating a quantitative pattern associated with “health” as with measurements in wethers that are not infected with *H. contortus*.

Although assumed to be sterile and generally void of other types of organisms^14,15^, blood does contain microorganisms without inducing disease^14,15^. Here, we purpose the use of a noninvasive, quantitative, blood-based method of assessing wether “health” that resembles the profile of those that are not infected with *H. contortus*.

Blood and gut (or ruminal) microorganisms are not directly reflective of each other. A significantly higher α-diversity (showing a larger variety or complexity of bacteria) are present in the gut than in blood^16^. A variety of known diseases previously considered non-communicable have recently been shown to cause an impact on health. These microbes are not easily culturable, therefore difficult to identify. Bacterial translocation into blood has been recognized to be responsible for infectious as well as non-infectious disease states^16–19^.

It is being stated here that different types of stimuli associated with inflammation patterns exist with blood microbiota as they do with gut microbiota. Therefore, although blood and rumen microbiome are not reflective of one another, an observable pattern of blood microbiota is indeed present, albeit it is assumed to be a much lower quantitative pattern^18^. Visual verifications of large numbers of adult *H. contortus* were made for all infected treatments during this study.

We hypothesize that blood-based tools can be used as prognostic, diagnostic, and therapeutic tools able to quantitatively identify “health” or “sickness” when exposed to *H. contortus* parasitism. We use transcript and metagenomic analysis in movement towards development of quantitative, blood-based tools. We hypothesize; (i) that the bacterial composition of the microbiome in wethers becomes altered during parasite infection; (ii) that the response is expressed through transcripts exhibited by the host genome and its microbiome; (iii) that there would be a visibly apparent quantitative pattern differentiating controls versus treated types; (iv) parasites affect host transcripts and host microbiomes; (v) that health can be associated with controls through indications of the deviation from a quantitative threshold of the *Firmicutes*/*Bacteriodetes* (*F/B*) ratio; and (vi) that the bacterial diversity/richness in wethers would change to resemble “health” as they age.

## Objectives

The objective of this study is to investigate the possibility of a noninvasive, quantitative, blood-based method to be used as a diagnostic, prognostic, and therapeutic tool for *H. contortus* parasite infection, detection, and prevention.

## Results

The initial experiment was organized as follows for each data group 7 dpi and 21 dpi: 5 wethers were not infected with *H. contortus* (controls), 5 received infection with *H. contortus* only (treated), 5 were infected with *H. contortus* and received ZA injections (ZA), and 5 were infected with *H. contortus* and received AB injection (AB). In this study transcript and microbiome analyses were completed. To characterize the nematode-infected host transcript and microbiome, we analyzed transcript patterns from 7 dpi (n = 19) and the microbiome portions at 7 dpi (n = 18) and 21 dpi (n = 20) (Supplementary Tables S1 and S2).

When using a numeric triad of p-values for controls (No Infection) versus treated (infection only), controls (No Infection) versus ZA (infection ZA inject), and controls (No Infection) versus AB (infection AB inject), a 95% confidence interval (* = p < 0.05) indicated likely significant changes in the expression of at least 184 transcripts (points selected) samples when ZA versus AB.

**Figure 1:**
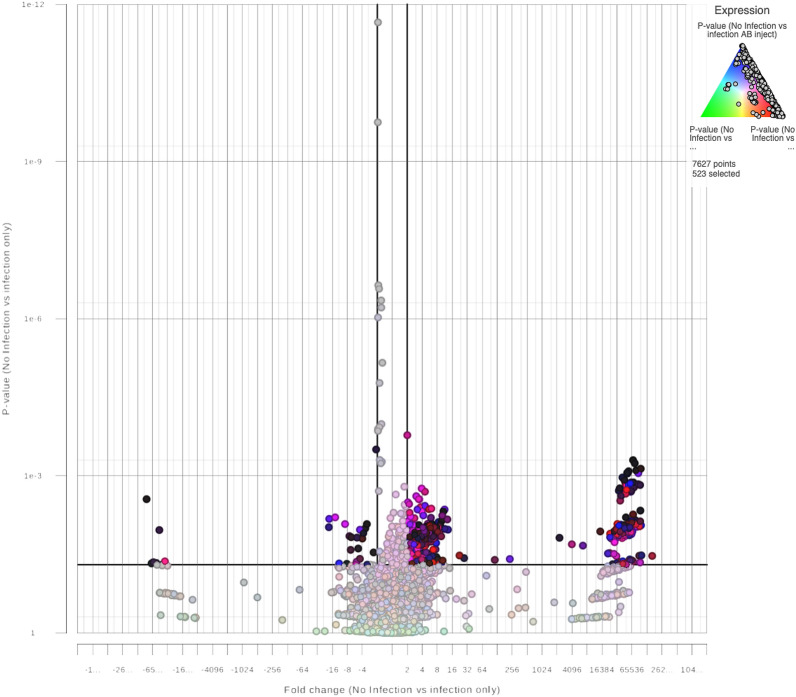
Controls vs treated. A Volcano plot for 19 blood samples indicating likely significant expression of transcripts (* = p < 0.05) based on treatment type of *Capra hircus* following STAR alignment and GSA differential analysis for transcript sequences of 7,627 identified transcripts (points selected) on 7 dpi. The fold change indicates downregulation (* =  < − 2), no change (NC; * =  > − 2, * =  < 2), and upregulation (* =  > 2) transcript distribution when comparing control samples to treatment samples as expressed against a Numeric Triad of P-values for controls vs treated (green), controls vs ZA (red) and controls vs AB (blue).

Volcano plots (showed transcripts that were identified as downregulated (* =  < − 2), having no fold change (NC: * =  > − 2, * =  < 2), and/or identified as upregulated (* =  > 2). Volcano plots identified related distributions of transcripts out of 7,627 expressed. The distribution was dependent on the wether (subject) and treatment type. The distribution of transcripts was based on comparisons using a numeric triad of p-values for controls versus treated. Controls versus (vs) ZA, and controls vs AB. Significance was based on a 95% confidence interval (* = p < 0.05) where fold changes of downregulation, NC, and upregulation transcript distributions of subject and treatment type were assessed. Comparison was made between controls vs treated (Fig. 1). Transcripts (points selected) numbering 523 were significant in downregulation, NC, and upregulation. The comparison of controls (No Infection) vs ZA, 290 transcripts (points selected) were significant. A comparison of controls vs AB showed 289 transcripts that were significant. In comparison of treated (infection only) vs ZA, identification of 338 transcripts with significance resulted. Additional Volcano plots and the list of genes and fold changes associated for all plots are available as supplemental data (Supplementary Figs. S1, S2, S3 and S4).

### Blood metagenomic composition

The average number of raw reads for 16S rRNA analyses of blood samples from hosts using Kraken and Mothur (v1.45.3) were compared. The distribution of raw read counts indicated the apparent difference in number using Kraken between 7 dpi (n = 18) and 21 dpi (n = 20). Kraken showed that there were 43.61 raw reads for metagenomic analyses of blood collected on 7 dpi as compared to 2,638.80 raw reads for metagenomic analysis of blood samples at 21 dpi.

The average distribution of raw read counts for 16S rRNA analysis of blood samples from hosts using Mothur (v1.45.3) identified an average distribution to vary between 7 dpi (8,926 raw reads per blood sample, (n = 18)) and 21 dpi (183,457 raw reads per blood sample (n = 20)) (Supplementary Table S4).

We examined how different treatments affect levels of alpha diversity, and how such levels progress within a certain treatment with time using Kraken and Mothur. Comparing the Shannon diversity indices between all four conditions at 7 dpi shows that significant differences between 7 and 21 dpi microbial composition exist for all comparisons using Kraken (Supplementary Tables S1 and S2). Simpson index for comparison of control and AB treatment also indicate significance with AB treatment subjects having more evenness in bacterial groups when compared to controls. A similar comparison between all four conditions at 21 dpi showed that there is only one significant change (No Infection vs ZA) in Shannon and Simpson diversity index using Kraken (Supplementary Table S2). Collectively levels of alpha diversity vary between AB and controls early (7 dpi) but not late (21 dpi) when using Mothur for analysis (Supplementary Table S3). Whereas collective levels of alpha diversity appear to vary between AB and controls and ZA and controls for both early and late indices when using Kraken for analysis (Supplementary Tables S1 and S2).

Comparison of diversity indices within a same treatment at 7 dpi and 21 dpi shows statistically significant difference in Shannon diversity where infected only is compared to ZA (Supplementary Tables S1 and S2). The diversity of both infected only and ZA subjects doubled at 21 dpi when compared to the same treatment at 7 dpi. Alternatively, the Simpson index was found to be significant when the controls at 7 dpi were compared to controls at 21 dpi. The controls at 21 dpi were found to have threefold more richness/evenness compared to controls at 7 dpi. Collectively this shows that alpha diversity for ZA increases with time in samples exposed to *H. contortus* infection (Supplementary Table S2).

**Figure 2:**
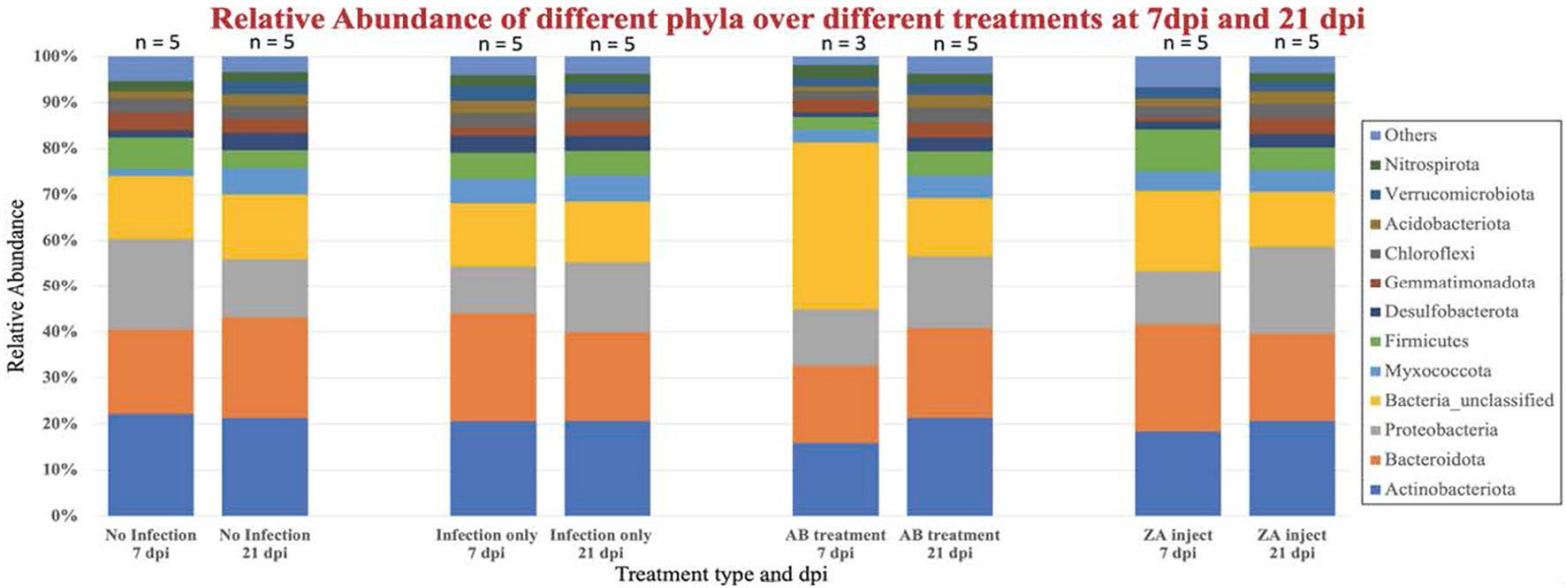
Relative Abundance of different phyla at 7 dpi and 21 dpi with 4 different treatments.

16S rRNA Relative Abundance profiles (Fig. 2) showed that for all 7 dpi samples combined, the highest mean relative abundant phyla were Actinobacteriota (19.23%) followed by Bacteroidota (20.5%), Proteobacteria (16.5%), and Bacteria unclassified (12.35%). Whereas for 21 dpi the most abundant phylum was Actinobacteriota (21%) followed by Bacteroidota (19.9%), Proteobacteria (15.9%), and Bacteria unclassified (13%).

We examined how different treatments affect community structure profiles, and how microbial community progresses within a certain treatment with time. We identified differences in microbial community profiles across various conditions in 7 dpi samples, as well as in 21 dpi samples (Supplementary Table S3). In infection only subjects the application Mothur was used to identify that there was an increase in the relative abundance of *Proteobacteria* (5%) and a decrease in *Bacteriodata* (4.1%) over time when comparing 7 dpi and 21 dpi subjects (Supplementary Table S3). For AB treatments, the percentage abundance of *Actinobacteria* increased by 6% and *Proteobacteria* also increased by 3.8% whereas the bacteria unclassified increased by 10.4% when examining control subjects. Finally, for ZA subjects, there were increases of Proteobacteria (7.53%), bacteria unclassified (5.6%), Actinobacteria (2.27%), and Gemmatimonadetes by 2.57%; however, there was a decrease in Bacteroidota (4.33%), (Supplementary Table S3).

Comparison between treatments at 21 dpi showed relatively smaller shifts in bacterial community composition. There was an increase in Proteobacteria (by 3% in infection only as well as AB treatment and by 7% in ZA) and decrease in unclassified bacteria (by 1% in infection only as well as AB and 2% in ZA) when compared to control subjects. Collectively these results suggest that specific shifts occur in response to infection, AB and ZA at both 7 dpi and 21 dpi samples (Supplementary Table S3). Therefore, when monitoring temporal progression of microbial communities within a specific condition, interesting patterns emerged. This shows that the progression of specific bacterial phyla changes over time with differing treatments. Collectively this shows differences between early and late stage of infection (Figs. 1, 2; Supplementary Table S3).

Of special importance is the use of Mothur to obtain *Firmicutes*/*Bacteroidetes* (F/B) ratios, since the ratios have been linked to *H. contortus* parasite infection^19^ and related to general health and dysbiosis in ruminants (PMC54041910)^21–23^. In the control group (Not Infected), the F/B ratio was 0.38 at 7 dpi and decreased to 0.18 at 21 dpi (Table 1). This ratio for control at 21 dpi (0.18) is lower than that observed in all other treatments at 21 dpi (Infection only = 0.28, AB = 0.27 and ZA = 0.26) (Supplementary Table S4). In contrast, infection induced an opposite trend: an initial decrease in F/B ratio to 0.24 at 7 dpi but subsequently increased to 0.28 at 21 dpi. The F/B ratio profile in ZA followed a similar pattern to no infection whereas the AB has F/B profile trend more like infection only (Table 1). This suggests that the AB bring the F/B profile closer to uninfected F/B profile when compared to infected or ZA samples. These values support the idea that measurable outcomes that can differentiate metabolically healthy wethers from parasitically compromised wethers based on changes in *Bacteriodetes* % Composition to *Firmicutes* % Composition^17–19^.

**Table 1:** Average F/B values for different treatment samples at 7 dpi and 21 dpi.

Treatment\Dpi	7 dpi	21 dpi
No Infection	0.38	0.18
Infection only	0.24	0.28
AB treatment	0.17	0.27
ZA inject	0.39	0.26

## Discussion

To characterize the nematode-infected host transcript and microbiome, we examined transcript patterns from 7 dpi (n = 19) and the microbiome at 7 dpi (n = 18) and 21 dpi (n = 20). We focused on whole-blood or buffy coat derived extractions of blood-based samples^24–26,31,32^ to discriminate among controls and treatments. We performed transcript analysis using STAR^20^, completing differential analysis using GSA^21^ and compared tools for microbial assignments between Kraken^22^ and Mothur (v1.45.3). One transcript analysis identified 7627 gene points selected that were expressed and show differences in association across treatment types at 7 dpi.

Unlike the results described by a previous study where it was surmised that infection by *H. contortus* did not affect caprine microbial diversity^5^, we identified that in goat blood samples there are changes in diversity and richness^28^. Microbial composition changes in diversity and richness over age (dpi), even within the interval of a few days^32–35^, further supporting that microbial flora diversity and richness are associated with health or the indication of no infection^30,31^ with age. We identified that there is an existence of likely significance based especially on treatment types. We also determined that blood-based metagenomic analysis can be used to identify differences between healthy controls and treatments of *Capra hircus* wethers that are infected with *Haemonchus contortus*^27,31^.

Factors, including antibiotics, can change the composition of microbiota^36^ often destroying the composition of beneficial microbes along with pathological ones. Thus, causing dysbiosis or the development of unwanted microbes^29,33^. Thus, the residual infection condition is still evident despite antibiotic or other types of treatment, indicated by inflammatory results or an increase in ratio of inflammation causing microbial flora (*Firmicutes*). We identified evidence that the composition of inflammatory microbiota increases with ZA or AB treatment. With a greater increase being exhibited by AB.

Our results imply that there are differences in transcripts as subjects become older or are exposed to parasitic infection longer. Attributes of the different treatment types show that transcripts are expressed in response to the *H. contortus* presence. All pointing towards the implication of *H. contortus* to effectively change and disrupt the internal habitat of the host.

Health may be determined based on *Firmicutes*/*Bacteroidetes (F*/*B)* ratios. The *F*/*B* Ratio is estimated by utilizing the lowest and highest values of the non-infected reference range (controls)^18^. When the value of *F*/*B* falls above a threshold, it would be considered in the “high” range, whereas falling below a threshold, would be considered in the “low” range^18^. Therefore, “health” is determined by where the *F*/*B* ratio lies, being quantitatively drawn from ratios of non-infected ranges of reference.

A high *F*/*B* ratio may be related to increased caloric extraction from food, fat deposition and lipogenesis, impaired insulin sensitivity, and increased inflammation. An elevated comparison to the reference range of harmful intrusion, may indicate that a hyperactive or inflamed response is occurring due to parasitic burden. Whereas a low *F*/*B* ratio, may be an indicator of dysbiosis. A ratio that is depressed compared to the reference range of harmful intrusion, may indicate that the host does not have adequate means of defending itself, thus the parasite load is depleting host resources. Both measures are postulated to correlate to decreased diversity of the microbiome compared to healthy non-infected cohorts (controls).

## Conclusions

We hypothesized that blood-based tools can be used to identify resistance to *H. contortus* parasitism. We used transcripts and metagenomic analysis towards developing blood-based tools. We identified that the bacterial composition of the metagenome in wethers becomes altered during parasite infection. We showed that the response is expressed through transcripts exhibited by the host genome and its metagenome. There is a visibly apparent quantitative pattern differentiating controls versus treated subjects. The host genome and microbiome respond together to parasitic invasion. The elevation/depression of the *Firmicutes*/*Bacteriodetes* (*F/*B) ratio indicates “health” or similarity to controls. We finally identified that bacterial diversity/richness in wethers changes to resemble “health” as they age.

The metabolic systems affected warrant further investigation to identify specific pathways where significant changes have resulted based on being exposed to infection. Specific transcripts may also require further in-depth study to determine specific functionality. Furthermore, the development of computational algorithms for correlation of microbial abundance and diversity are warranted. The authors conjecture that blood samples are shown here to be a possible means to indicate *H. contortus* infection based on detection of microbial flora abundance and diversity as well as in transcript profiles. Correlations can be drawn on statistical levels of microbial flora for this specific type of inflammation. The specificity in the range of microbial flora may indicate the occurrence of depleting resources or inflammation due to *H. contortus* infection. In other words, this implies metagenomic and distant transcriptional effects by *H. contortus* that changes and disrupts the health of the host with measurable quantitative results. The development of a standard laboratory diagnostic procedure using blood microbiota to detect gastrointestinal infection with *H. contortus* is the ideal course of action.

## Materials and methods

Forty wethers (10 per treatment) were used as non-infected controls, others infected with *Haemonchus contortus* and anti-γδ T-cell antibodies*,* also some infected with zolodronic acid (a bisphosphonate derivative), all under controlled conditions. We evaluated wethers following the introduction of infections at 7 dpi and 21 dpi. Vitals were examined through weight check, whole blood evaluation through CBC with Differential Count if indicated, and FEC tested. Expression of 7627 genes were identified through different treatment types after 7 dpi. Testing and evaluation were completed through extraction of DNA of the samples of blood utilizing modified protocols of Microbiome DNA Isolation Kit from Norgen Biotek Corp. (Thorold, ON, Canada). Reverse transcribed cDNA libraries were constructed and later sequenced using Swift Biosciences kits. Analysis was evaluated through default Partek Flow software suites (St. Louis, Missouri, USA) for microbiomes using Kraken at 7 dpi (n = 18) and 21 dpi (n = 20). Blood samples datasets were obtained through sequencing using the NGS Illumina RNA-Seq instrument. Quality assurance and quality control analytics were used for 7 dpi (n = 19). In all there were five goat wethers in four treatment groups for the analysis of this research. So, the CBC with Diff Count if indicated and other blood test can distinguish certain markers to notify health and other immune response symptoms.

### Animals and treatments

Forty Alpine wethers (114.2 ± 0.92 d of age and 19.4 ± 0.33 kg BW at the start of the experiment) that had been raised in indoor pens at the Langston University Research Farm were used. The wethers were checked for fecal egg counts (FECs) and confirmed to be nematode-parasite free. Whethers were placed in pens by BW and then randomly allocated to treatments within each pen. Thereafter, a small number of treatment assignments were changed to achieve more similar mean and variation in BW. There were no exclusions. All animals were allowed to acclimatize to pens and feeders for daily supplies of 200 g concentrate pellet per animal composed of 500 g of ground grass (50%) and alfalfa (50%) hays. The treatment groups were as designed in Table 2.

**Table 2:** Experimental set-up. Treatment Group (1–4) for infection L3 *H. contortus* (+) or non-infection *H. contortus* (−), with (+) or without (−) treatment type (Zoledronic acid (ZA) injection or γδ T depletion (AB)).

Group	L3 *H. contortus* infection	Zoledronic acid	γδ T depletion
1	−	−	−
2	+	−	−
3	+	+	−
4	+	−	+

On the first (1) day prior to the L3 infection, the AB injection was administered intravenously. ZA were administered intravenously 7 days prior to and 0, 7, and 14 days after the L3 infection. At the beginning of the experiment all kids except Group 1 were given 10,000 *H. contortus* infective larvae (L3; hatched and isolated from feces being collected from LU goats) by gavage. Five animals from each group were euthanized on 7 dpi for sampling and the other five animals were euthanized 21 dpi.

### Blood sample collection and processing

Blood samples were collected from five goats in four treatment groups 7 dpi. Blood samples included red blood cells, white blood cells (total and differential), hemoglobin, platelets, and plasma protein, from the jugular vein were collected in EDTA tubes. Quality assurance/quality control (QA/QC) parameters resulted in blood samples from 19 cDNA libraries that were used from samples collected 7 dpi. The cDNA libraries were sequenced on an Illumina RNA-Seq Next-generation sequencing (NGS) instrument and filtered and normalized using default Partek Flow software suites (St. Louis, Missouri, USA).

Methods of identifying naturally occurring microbial flora in nontreated and treated wethers were identified. Profiles are based on primers spanning the hypervariable regions V1–V9 of the 16S rRNA gene and the internal transcribed spacer (ITS) region 1 amplified with primers ITS1 and ITS2. PCR reactions consisted of 50 ng input DNA per reaction completed to 20 μL reaction mixture. Each sample was amplified with random barcoded primer combinations. Reactions were quantified with a NanoDrop™ 2000 (Thermo Fisher Scientific, Waltham, MA 02451). Analytics for QA/QC for high-throughput barcoded Illumina MiSeq NGS sequencing of 16S rRNA, resulted in 18 samples that were obtained for 7 dpi and 20 samples for 21 dpi.

### Total RNA purification

Total RNA was collected from blood samples using a modified TRIzol reagent procedure (Thermo Fisher Scientific, Waltham, MA, USA).

### Lysate preparation from blood

The cDNA libraries were constructed by initial first strand synthesis using the Protocol for Non-directional RNA-seq Workflows and NEBNEXT (New England Biolabs, Ipswich, Massachusetts, USA) Ultra II RNA First Strand Synthesis Module (E7771), according to a modified manufacturer’s protocol.

### First strand cDNA synthesis reaction

The first strand synthesis reaction was assembled on ice by adding components to the fragmented and primed RNA for a total volume of 20 mL. The reaction was mixed thoroughly by pipetting. The sample was incubated in a preheated thermocycler with the heated lid set at 80 °C as follows: Step 1: 10 min at 25 °C; Step 2: 15 min at 42 °C; Step 3: 15 min at 70 °C; and Step 4: Hold at 4 °C. We then proceeded directly to Swift Biosciences (Ann Arbor, Michigan, USA) ACCEL-NGS 1S PLUS DNA LIBRARY KIT: Single, Dual Combinatorial and Unique Dual Indexing and prepared the DNA Libraries.

### Microbiome DNA isolation

Microbiome DNA was collected from blood samples using a modified Microbiome DNA Isolation Kit from NORGEN BIOTEK CORP. (Thorold, ON, Canada).

### Sequencing

Quality Assurance/Quality Control of bar-coded sequence prepped samples of cDNA and the cDNA library sequencing with an Illumina RNASeq NGS instrument were completed by the Genomics Core Facility at Oklahoma State University (Stillwater, Oklahoma, USA).

Quality Assurance/Quality Control of bar-coded sequence prepped samples were completed and sequenced for 16S rRNA metagenomics of blood samples by Swift Biosciences (Ann Arbor, Michigan, USA) using an Illumina MiSeq NGS instrument.

### Computational analysis

Several methods were utilized to conduct bioinformatic analysis of the obtained sequence data. For gene expression analyses, we used the Partek Flow (St. Louis, Missouri, USA) software suites pipelines that include, but are not limited to the STAR algorithm, Normalization, and the gene set differential analysis method (GSA).

Preliminary analyses included Qiime2 open-source analysis for the metagenomic or microbiome analysis. The results obtained utilized the Kraken pipeline through the Partek Flow (St. Louis, Missouri, USA) software suites. Inflammation comparisons were based on the ratio of *F*/*B* ratios that are evident as well as the reduction of microbial diversity.

Mothur (v1.45.3) was also used for microbiome analysis. The MiSeq manual protocol (http://mothur.org/wiki/miseq_sop/) was followed to obtain the 16S rRNA diversity, classify sequences into OTUs and calculate alpha diversity index such as Shannon diversity index and Simpson index. For Shannon and Simpson diversity index values, the student t-test was calculated, and the significance of these indices were based on p-value of < 0.05.

### Ethical approval and consent to participate

The treatment of animals is abided by the guidelines of the Langston University Institutional Animal Care and Use Committee (LUACUC) Approval # 2018-14. All experiments were performed in accordance with relevant guidelines and regulations. Furthermore, the reporting in this manuscript is in accordance with ARRIVE guidelines.

### Consent for publication

All authors have consented for publication.

## Supplementary Information

Supplementary Legends.

Supplementary Figure S1.

Supplementary Figure S2.

Supplementary Figure S3.

Supplementary Figure S4.

Supplementary Tables.

## Data Availability

The metagenomic data have been deposited with links to BioProject accession number PRJNA612987 in the NCBI BioProject database (https://www.ncbi.nlm.nih.gov/bioproject/). The gene expression data discussed in this publication have been deposited in NCBI's Gene Expression Omnibus^37^ and are accessible through GEO Series accession number GSE169607 (https://www.ncbi.nlm.nih.gov/geo/query/acc.cgi?acc=GSE169607.
