# Supplementary material for: Transcript and blood-microbiome analysis towards a blood diagnostic tool for goats affected by Haemonchus contortus
Source: Sci Rep. 2022 Mar 30;12:5362. doi: 10.1038/s41598-022-08939-x (PMC8967894; doi:10.1038/s41598-022-08939-x)
Supplement: Supplementary file 1 — Supplementary Legends. [file 41598_2022_8939_MOESM1_ESM.pdf]

Legends for Supplementary Tables

Transcript and blood-microbiome analysis towards a blood diagnostic tool for goats affected by *Haemonchus contortus*

Yonathan Tilahun[a,*], Jessica Quijada Pinango[a], Felicia Johnson[a], Charles Lett[a], Kayla Smith[a], Terry Gipson[a], Malcolm McCallum[a], Peter Hoyt[b], Andrew Tritt[c], Archana Yadav[d], Mostafa Elshahed[d], and Zaisen Wang[a]

Supplementary Table S1. Average of Shannon and Simpson indices results for 7 dpi using Kraken.

Supplementary Table S2. Average of Shannon and Simpson indices results for 21 dpi. using Kraken.

Supplementary Table S3. Average number of sequences over different treatments at 7 dpi and 21 dpi using Mothur.

Supplementary Table S4. Raw number of sequences in samples for 21 dpi. The Mothur calculated *Firmicutes*/*Bacteroidetes (F/B)* ratios.
