# Supplementary material for: Transcript and blood-microbiome analysis towards a blood diagnostic tool for goats affected by Haemonchus contortus
Source: Sci Rep. 2022 Mar 30;12:5362. doi: 10.1038/s41598-022-08939-x (PMC8967894; doi:10.1038/s41598-022-08939-x)
Supplement: Supplementary file 3 — Supplementary Figure S2. [file 41598_2022_8939_MOESM3_ESM.docx]

**Legends and Supplementary Figures**


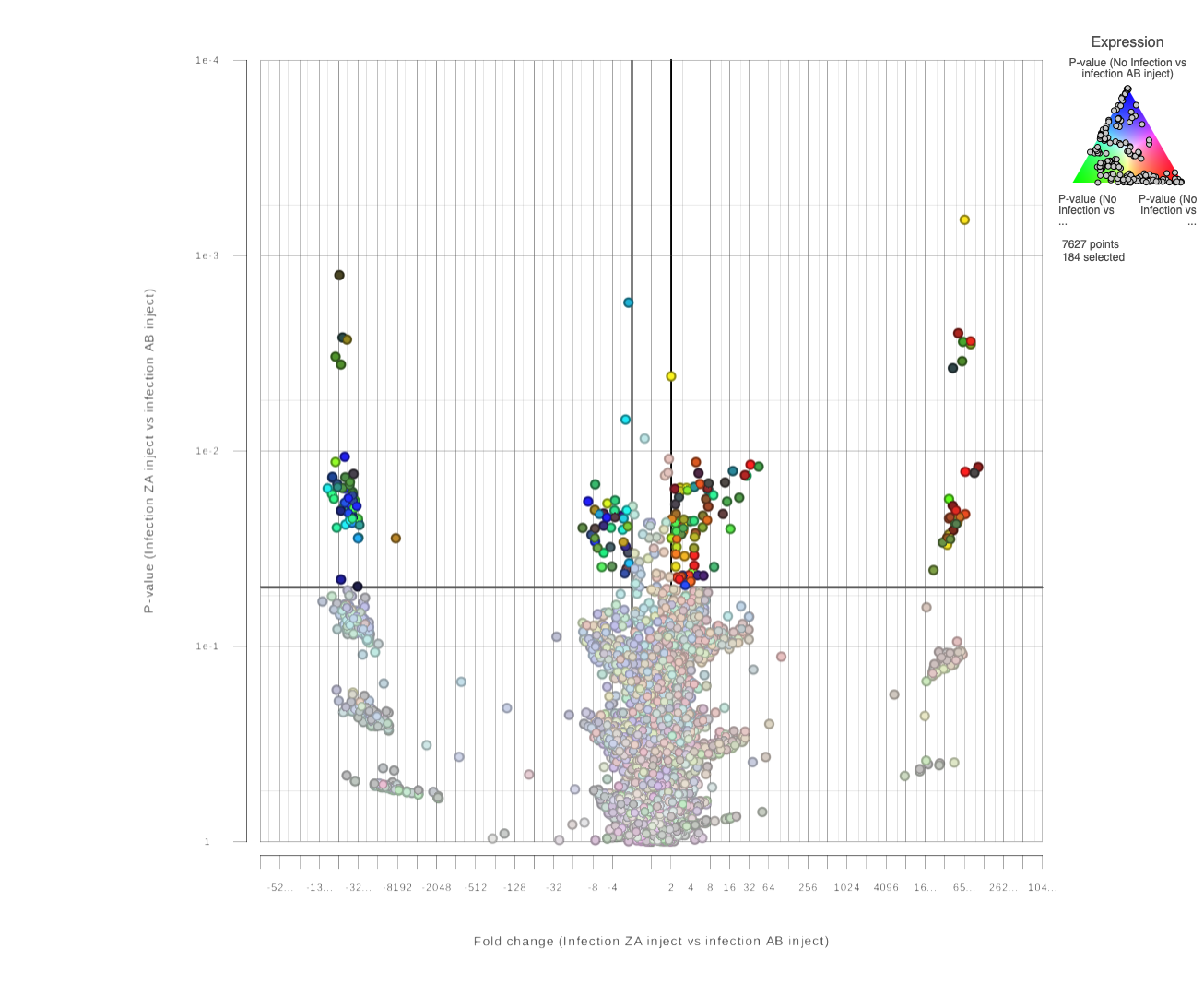


Supplementary Figure S2. A Volcano plot for 19/20 blood samples indicating likely significant expression of genes (* = p < 0.05) based on treatment type of *Capra hircus* following STAR alignment and GSA differential analysis for transcript sequences of 7627 identified genes expressed on 7 dpi. The fold change indicates downregulated (* = < -2), no change (NC; * = > -2, * = < 2), and upregulated (* = > 2) gene distribution when comparing Infection ZA inject samples to infection AB inject samples when expressed against a Numeric Triad of P-values for No Infection vs Infection only (green), No Infection vs Infection ZA inject (red) and No Infection vs infection AB inject (blue).
