# Supplementary material for: Transcript and blood-microbiome analysis towards a blood diagnostic tool for goats affected by Haemonchus contortus
Source: Sci Rep. 2022 Mar 30;12:5362. doi: 10.1038/s41598-022-08939-x (PMC8967894; doi:10.1038/s41598-022-08939-x)
Supplement: Supplementary file 4 — Supplementary Figure S3. [file 41598_2022_8939_MOESM4_ESM.docx]

**Legends and Supplementary Figures**


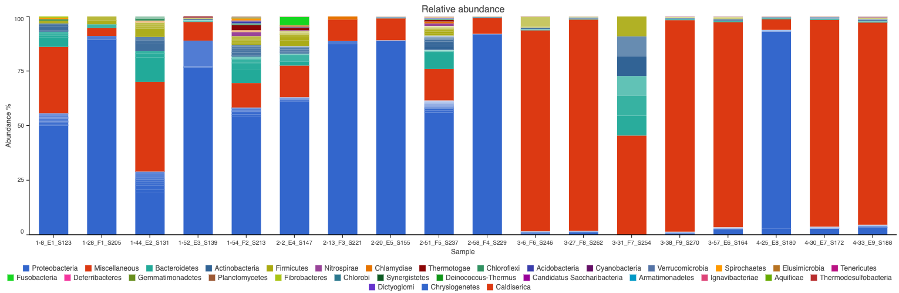


**Supplementary Figure S3. Relative Abundance of OTUs at 7 dpi for 18 samples.** The first number before each sample ID identifies the treatment type (1 = Control, 2 = Treated, 3 = ZA, and 4 = AB).
